# Is platelet-rich plasma better than steroids as epidural drug of choice in lumbar disc disease with radiculopathy? Meta-analysis of randomized controlled trials

**DOI:** 10.3389/ebm.2025.10390

**Published:** 2025-02-04

**Authors:** Sathish Muthu, Vibhu Krishnan Viswanathan, Prakash Gangadaran

**Affiliations:** ^1^ Department of Spine Surgery, Orthopaedic Research Group, Coimbatore, India; ^2^ Department of Biotechnology, Faculty of Engineering, Karpagam Academy of Higher Education, Coimbatore, India; ^3^ Department of Orthopaedics, Government Medical College, Karur, India; ^4^ Department of Orthopaedics, Devadoss Hospital, Madurai, India; ^5^ Department of Nuclear Medicine, School of Medicine, Kyungpook National University, Daegu, Republic of Korea; ^6^ BK21 FOUR KNU Convergence Educational Program of Biomedical Sciences for Creative Future Talents, School of Medicine, Kyungpook National University, Daegu, Republic of Korea; ^7^ Cardiovascular Research Institute, Kyungpook National University, Daegu, Republic of Korea

**Keywords:** PRP, epidural steroid, degenerative disc disease, pain relief, biologics

## Abstract

The current meta-analysis was performed to analyze the efficacy and safety of platelet-rich plasma (PRP) as an epidural injectate, in comparison with steroids in the management of radiculopathy due to lumbar disc disease (LDD). We conducted independent and duplicate searches of the electronic databases (PubMed, Embase and Cochrane Library) in March 2024 to identify randomized controlled trials (RCTs) analyzing the efficacy of epidural PRP for pain relief in the management of LDD. Animal or *in vitro* studies, clinical studies without a comparator group, and retrospective or non-randomised clinical studies were excluded. Diverse post-intervention pain scores [visual analog score (VAS)] and functional scores [Oswestry Disability Index (ODI), SF-36], as reported in the reviewed studies, were evaluated. Statistical analysis was performed using STATA 17 software. 5 RCTs including 310 patients (PRP/Steroids = 153/157) were included in the analysis. The included studies compared the efficacy and safety of epidural PRP and steroids at various time-points including 1, 3, 6, 12, 24, and 48 weeks. Epidural PRP injection was found to offer comparable pain relief (VAS; WMD = −0.09, 95% CI [−0.66, 0.47], p = 0.641; I^2^ = 96.72%, p < 0.001), functional improvement (ODI; WMD = 0.72, 95% CI [-6.81, 8.25], p = 0.524; I^2^ = 98.73%, p < 0.001), and overall health improvement (SF-36; WMD = 1.01, 95% CI [−1.14, 3.17], p = 0.224; I^2^ = 0.0%, p = 0.36) as epidural steroid injection (ESI) at all the observed time points in the included studies without any increase in adverse events or complications. Epidural administration of PRP offers comparable benefit as epidural steroid injection (ESI) in the management of radiculopathy due to LDD. The safety profile of the epidural PRP is also similar to ESI.

## Impact statement

This manuscript makes an important contribution to the field by providing a comprehensive meta-analysis on the use of platelet-rich plasma (PRP) as an alternative to steroids for epidural injections in managing radiculopathy caused by lumbar disc disease (LDD). By comparing PRP and steroids across multiple randomized controlled trials, this work advances the field by offering robust evidence that PRP provides similar pain relief, functional improvement, and overall health benefits as steroids, without increased risk of adverse events. This new information introduces a potential treatment option that could reduce dependency on steroids, thus having significant implications for patient care. The findings are timely and relevant, as they highlight PRP’s comparable efficacy and safety, potentially shifting clinical practice toward non-steroid-based interventions for managing LDD-related radiculopathy.

## Introduction

Lumbar radicular pain is a well-known cause for spinal disability secondary to mechanical compression of the nerve roots or inflammatory responses to the inciting stimuli [1, 2]. Conservative measures like bed rest, anti-inflammatory medications and physical therapy constitute the first line of management in these patients [3]. However, 20% of patients have recurrent or recalcitrant pain or symptoms despite such non-surgical measures [4].

Interlaminar or transforaminal epidural steroid injections (ESIs) have been acknowledged as interventions short of surgery, which may mitigate the symptoms and reduce the radicular pain [5, 6]. Traditionally, triamcinolone has remained the therapeutic drug utilized for epidural injections, in view of its excellent anti-inflammatory property and relatively low adverse events [7, 8]. While a majority of studies have reported substantial pain relief during the initial 3 months following ESI; the evidence in the current literature on the long-term outcome including need for surgical intervention at the end of 1 year is still largely unclear [9, 10]. In addition, certain studies have reported severe complications including allergic reactions, sepsis and chronic adrenal suppression [11, 12].

Platelet-rich plasma (PRP) is growingly recognized as an important adjuvant component in the field of orthopedic surgery, whose properties depend on the platelet and white blood cell (WBC) concentrations. Numerous cytokines within PRP like transforming growth factor (TGF) -β1, interleukin-1 receptor antagonist (IL-1RA), insulin-like growth factor (IGF-1) and platelet-derived growth factor (PDGF) form the basis for their regenerative and anti-inflammatory actions in the healing of various pathologies [13, 14]. In addition, in view of the autologous and antimicrobial nature of PRP, studies have indicated relatively lower concerns with regard to the infective and immunogenic complications following its use [15, 16]. Over the recent years, diverse studies have evaluated the role of PRP in the management of different degenerative, neuropathic and inflammatory pathologies of the spine [10, 17–19]. Even though meta-analyses have been published on the role of PRPs in spinal conditions, a majority of these reviews have followed inconsistent strategies for study inclusion; and have considered evidence from retrospective and non-randomized studies too [20–30]. In addition, may studies have also reviewed the role of PRP administration through different routes for a wide variety of spinal pathologies [16, 20]. As a result of such heterogeneity in the methodological quality and evidence available in the existing literature; diverse issues regarding the status of epidural administration of PRP in the management of lumbar radiculopathy, including its safety and efficacy (based on outcome measures like functional scores, improvement of pain scores, incidence of treatment failure and complication rates) are still largely controversial. The current meta-analysis was thus planned to comprehensively evaluate only the randomized controlled trials (RCTs) in available literature; and compare the safety profile and efficacy of epidural PRP injections with traditional ESIs.

## Materials and methods

This meta-analysis was performed in compliance with the recommendations of the Back Review Group of Cochrane Collaboration [25] and presented in adherence to the Preferred Reporting Items for Systematic Reviews and Meta-Analyses (PRISMA) statement [31].

### Search strategy

Two reviewers were involved in making an independent electronic literature search for RCTs evaluating the efficacy of PRP, as compared to steroids in the management of degenerative lumbar disc disease (LLD). The databases namely, PubMed, Embase and the Cochrane Library, were searched to identify all the relevant studies published until March 2024 (no specific date restrictions were applied to the search query).

**FIGURE 1:**
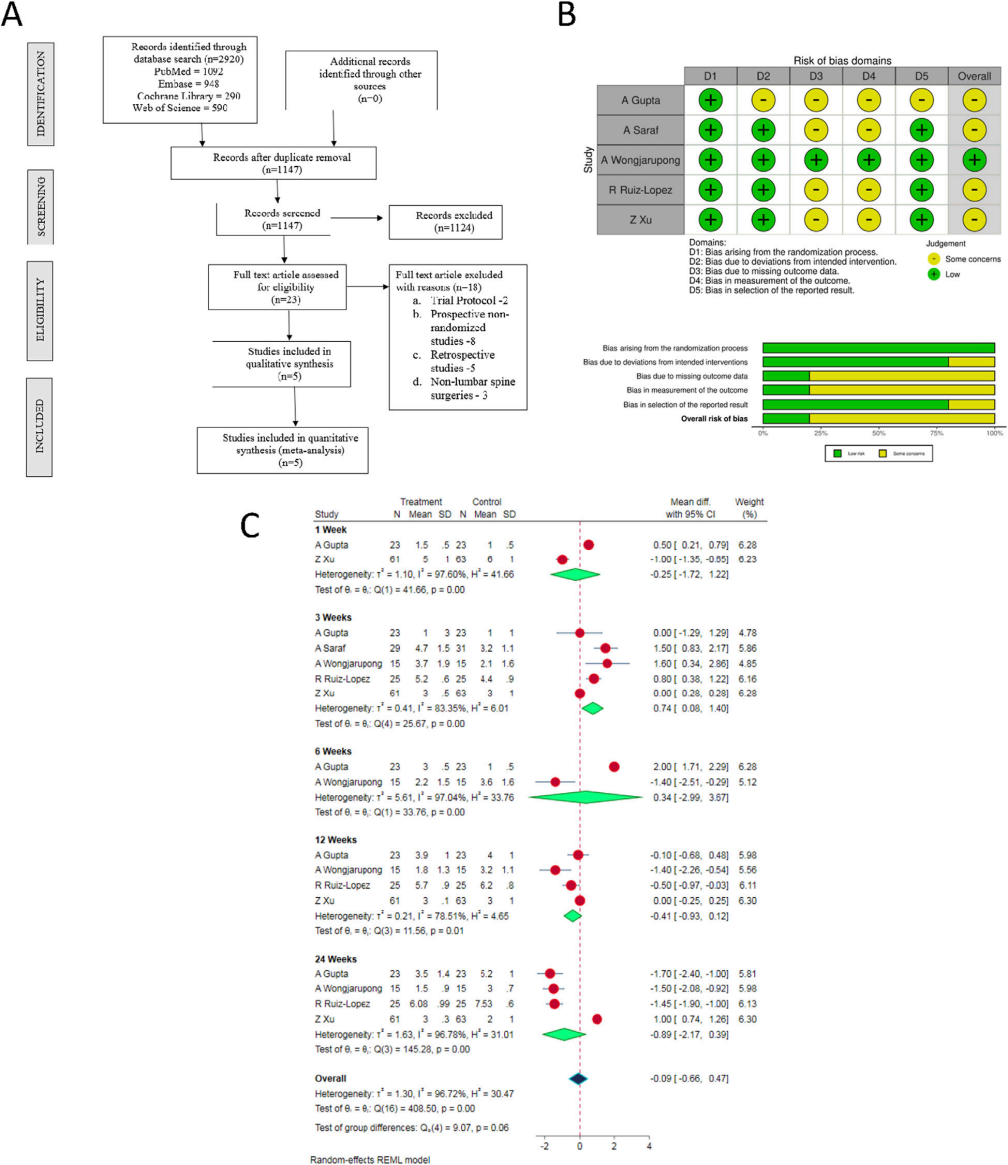
**(A)** PRISMA flow diagram of the included studies. **(B)** Quality and risk of bias assessment of all the included studies. **(C)** Forest plot of the included studies comparing post-operative pain score at various time points.

We used the following keywords in the database search: “Platelet-rich Plasma,” “Epidural steroid,” “Lumbar degenerative disc disease.” We also went through the references of the articles shortlisted from preliminary screening to identify studies missed in the preliminary search. Studies were then selected for the meta-analysis based on the specific inclusion and exclusion criteria, mentioned vide-infra. In case of any discrepancy in selecting the article, the final decision was made based on the consensus achieved through mutual discussions. The sequence of selecting the studies for the analysis has been shown in the PRISMA flow diagram (Figure 1A).

### Inclusion criteria

We included studies for analysis based on the PICOS criteria:

Population: Patients with lumbar disc disease with radicular pain.
Intervention: Epidural PRP.
Comparator: Epidural steroids.
Outcomes: Post-operative pain scores, functional scores, complications.
Study Design:  Randomized controlled trials (RCTs).

### Exclusion criteria

Studies were excluded from the review based upon the following criteria:

1. Animal studies involving PRP in disc disease conditions.
2.*In vitro* studies on PRP in disc disease models.
3. Studies without a comparator group such as case series and case reports.

### Data extraction

Two reviewers independently retrieved relevant data from the articles included for analysis. The following data were extracted from the reviewed studies:

 1. *Study characteristics*: Year of publication, authors, country, number of patients enrolled.
 2. *Baseline characteristics*: Mean age, gender proportions, levels involved, route of administration.
 3. *Primary Outcomes*: Post-operative pain scores
  *Secondary Outcomes*: Functional scores and overall health related scores.
 *Other Outcomes*: Adverse events and complications.

If any data was found missing from the included study, we contacted the corresponding authors of the study for necessary clarifications. All discrepancies were resolved through mutual discussions among the authors.

### Risk of bias and quality assessment

Two reviewers independently assessed the methodological quality of the included studies with the help of Cochrane Collaboration’s RoB 2 tool for RCTs with five domains of bias assessment included in them [32].

### Statistical analysis

We performed the meta-analysis of the pooled data in Stata software Version 17. In case of dichotomous variables, we utilised odds ratio (OR) with 95% Confidence Interval (CI). For analysing continuous variables, we used weighted mean difference (WMD) with 95% CI. We evaluated the heterogeneity of the pooled data using I^2^ statistics [33]. If I^2^ < 50% and p > 0.1, a fixed-effects model was employed in meta-analysis. On the other hand, if I^2^ > 50% and p < 0.1, random-effects model was utilised. Publication bias was evaluated with funnel plots and egger regression test. Heterogeneity was explored with galbraith plot. Further, subgroup and sensitivity analyses were performed to examine the causes of heterogeneity. A p-value of less than 0.05 was considered significant.

## Results

### Search results

Electronic database search resulted in 2,920 articles, which after initial screening through de-duplication, revealed a total of 1,147 articles. After title and abstract screening of these articles, 1,124 were excluded. 23 articles qualified for full-text review; among which, 18 were excluded. Finally, 5 RCTs [34, 35, 37, 38] involving a total of 310 patients (ESPB group/Control group = 153/157) were included in our meta-analysis. PRISMA flow diagram of study selection is given in Figure 1A. The general characteristics of the RCTs included in our analysis have been shown in Supplementary Table S1. PRP protocols of the included studies are shown in Supplementary Table S2.

### Quality assessment

The methodological quality of the included studies is depicted in Figure 1B. Based on the available data, none of the included studies had an overall high risk of bias which necessitated an exclusion from the analysis.

### Primary outcomes

#### VAS (visual analog scale) score

There were substantial variations in the time points at which the post-operative pain scores were evaluated in the included studies. Therefore, we considered only the post-operative pain score measurements at 1-, 3-, 6-, 12- and 24-week time points for our analysis (as reported in the individual studies). In view of the significant heterogeneity among the reviewed studies, random-effects model was utilized for analysis. Epidural PRP was shown to be comparable to the epidural steroid in relieving pain at all the observed time points with the overall [WMD = −0.09, 95% CI (−0.66, 0.47), p = 0.641; I^2^ = 96.72%, p < 0.001; as shown in Figure 1C].

#### ODI (oswestry disability index) score

**FIGURE 2:**
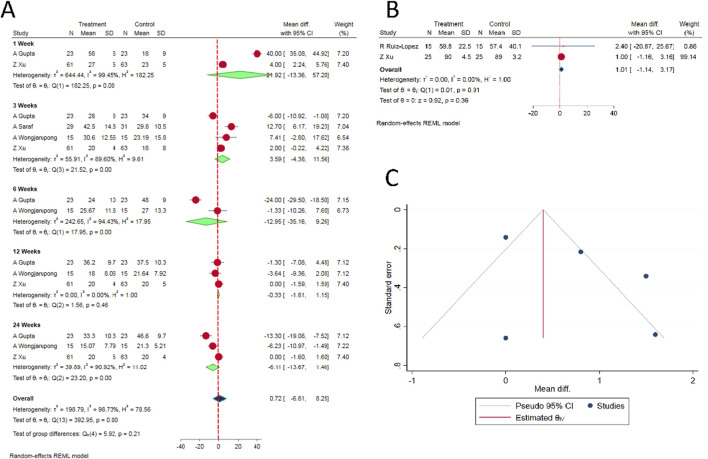
**(A)** Forest plot of the included studies comparing functional score at various time points. **(B)** Forest plot of the included studies comparing overall health score SF-36 at 6 months. **(C)** Funnel plot estimation of the publication bias in the included studies for analysis.

Similar to the VAS, there was substantial heterogeneity in the time points at which the ODI scores were measured in the included studies. Hence, we analyzed post-operative ODI scores reported at 1-, 3-, 6-, 12- and 24-week time points in the reviewed studies. There was no significant difference between the epidural PRP and epidural steroid injections with regard to the ODI scores at all the aforementioned time points [WMD = 0.72, 95% CI (−6.81, 8.25), p = 0.524; I^2^ = 98.73%, p < 0.001; as shown in Figure 2A].

#### SF-36 score

Based on our comparison of SF-36 scores between the epidural PRP and epidural steroid injections, there was no significant difference between the intervention groups at 6th month time point [WMD = 1.01, 95% CI (−1.14, 3.17), p = 0.224; I^2^ = 0.0%, p = 0.36; as shown in Figure 2B].

#### Complications

Based on our analysis of 3 studies, we could not observe any significant difference in the incidence of adverse events or complications between the epidural PRP and epidural steroid injection groups (p > 0.05) [34, 37, 38].

#### Publication bias analysis

Given the limited number of RCTs on the subject, estimating publication bias and sensitivity analysis to explore into the heterogeneity in the results among the included studies would not yield meaningful results. Nevertheless, funnel plot was generated for the VAS score for pain relief. We did not observe significant asymmetry as shown in Figure 2C. In addition, Egger’s regression test did not show significant publication bias (p = 0.274).

## Discussion

The overall prevalence of symptomatic low back pain (LBP) is approximately 1–3%; while the prevalence of lumbar radicular pain is reportedly around 0.98% [30, 35, 36]. Administration of medications through epidural route has been traditionally practised as an intervention short of surgery in patients with radicular symptoms secondary to lumbar spine pathologies like lumbar disc herniation (LDH) or canal stenosis [37]. Corticosteroids (especially triamcinolone) have quintessentially remained the drug of choice for epidural administration, although recent studies have demonstrated a lack of clear evidence regarding its superiority over placebo agents in improving function, obviating surgery or mitigating disability [12]. In addition, some researchers have also underscored the need to consider the numerous adverse events associated with steroid use, including its impact over diverse bodily functions [38]. It is well established that cytokine production from macrophages or disc cells play an important role in pain generation in patients with degenerative disc disease. In this context, the role of cell-based biological therapies such as autologous bone marrow concentrate (BMC), platelet rich plasma (PRP), mesenchymal stem cells (MSC), autologous conditioned serum (ACS) and platelet lysate in diverse spinal pathologies have been widely discussed [17, 19, 39, 40]. The current meta-analysis of RCTs was performed to comprehensively examine the role of PRP as an epidural injectate in mitigating LBP and lumbar radiculopathy.

### Rationale of biological agents in chronic LBP

The nucleus pulposus (NP) consists of diverse inflammatory cytokines and pain mediators including phospholipase A2, nitric oxide, prostaglandin E, and IL-1 [41, 42]. Studies have also demonstrated that disc material through inflammatory mediators produces direct chemical injury to the nerve root; and enhances the intra- and extra-neural inflammation, venous congestion as well as conduction block [43]. Among all the aforementioned mediators, IL-1 has been acknowledged to play a special role in the development of low back pain. Among the biological agents inhibiting IL-1, IL-1 receptor antagonist (IL-1RA), soluble IL-1 receptors, and type-1 cytokines like IL-4, IL-10, and IL-13 have been examined for their therapeutic efficacy.

With progressive developments in the biological agents, PRP and its derivatives, mesenchymal stem cells (MSC), plasma lysate, plasma rich in growth factors (PRGF-Endoret) and ACS have been proposed as biologics amenable to delivery through epidural route. ACS is a rich source of anti-inflammatory cytokines like IL-4, IL-10, IL-13, IL-1RA, fibroblast growth factor-2 (FGF-2), hepatocyte growth factor (HGF), and transforming growth factor-β (TGF-β) [39, 44]. Being an IL-1 receptor antagonist, ACS has been growing in popularity as an epidural treatment option in the form of a “biochemical sensitiser” of inflamed nerve roots [30, 40].

The platelet concentration within PRP is 3–8 times higher than the serum level, which facilitates its anti-inflammatory, angiogenic, cell-migration enhancing and anabolic potential for tissue regeneration. The activated platelets release diverse growth factors like TGF-β, insulin-like growth factor (IGF), platelet-derived growth factor (PDGF), vascular endothelial growth factor (VEGF) and fibroblast growth factor (FGF), which in turn, enable tissue healing through stimulation of chondrocytes, collagen synthesis, inhibition of cell apoptosis and regulation or catabolic cytokines. In addition, PRP has been shown to play a vital role in the healing of connective tissues like Sox9, AGN, COL I and COL II [23, 45].

### Preparations of PRP

The purification processes for PRP have broadly been categorised into open and closed techniques [20]. Diverse systems for centrifugation and concentration of platelets have been described in the literature; and different classification systems for such PRP preparations have been described. Certain basic and clinical studies have shown that high concentrations of leucocytes can negatively impact the PRP efficacy; however, the issue is still controversial across various indications [14, 46]. Dohan et al. [47] classified PRP preparations into pure-PRP (P-PRP), leucocyte-rich PRP (L-PRP) and pure platelet-rich fibrin (P-PRF), based on the relative concentrations of the components. Mishra et al. [48] further categorised PRP preparations in 8 categories based on leucocyte concentration, activation of leucocytes and platelet concentrations.

### Injection strategies in the published literature

Based on the review by Kawabata et al. [20], the role of PRP in the repair of degenerated disc, promotion of spinal fusion and enhancing neurological recovery after spinal cord injury (SCI) were discussed in detail. Studies have evaluated epidural injections through interlaminar (IL), transforaminal (TF) and caudal routes. Some studies also evaluated the role of PRP injections into paraspinal musculature (intra-muscular), facet joints, intravertebral disc space, sacroiliitis, and spinal ligaments [20, 26]. In a majority of the published studies, the injection was administered under fluoroscopic guidance, while ultrasound- and computed tomography (CT)-guided approaches were utilised in certain studies. A majority of the reviewed studies evaluated outcome following single-time PRP injection [14, 20].

### Results of our meta-analysis

Our meta-analysis is the first study to only evaluate all the RCTs hitherto published comparing the efficacy and safety of epidural corticosteroid and PRP injections for lumbar radiculopathy. All the previous systematic reviews or meta-analyses have included retrospective, non-randomised studies, studies involving other control arms for comparative analysis; or included studies evaluating different types of injections for LBP secondary to multiple spinal pathologies [20, 23, 26, 29, 30]. In view of such wide variations in the study designs and analysis, there is still substantial ambiguity in our knowledge regarding this subject and heterogeneity in the available results. Our meta-analysis was thus planned to examine the true role of this modality in the context of lumbar radiculopathy.

### Evidence from available RCTs

In the RCT by Ruiz-Lopez and Tsai (2020) [21], caudal injection of PRP in 50 patients with LBP provided substantial improvement in pain and disability during the immediate post-intervention period. The procedure also resulted in superior outcome than corticosteroids at the 6th month followup time point [49]. In recent double-blind, prospective, randomised controlled study (involving 46 patients) comparing transforaminal epidural PRP and corticosteroid injections, Gupta et al. [25] concluded that transforaminally-administered epidural PRP injection had significantly better outcome than steroid injection at the 6th week and 6th month time points; however, the outcome at the end of 1 year was comparable between the two groups.

In another recently-published RCT by Saraf et al. [24], substantially better outcome was observed in the steroid group at the end of 1 month (p < 0.001 for both VAS and modified ODI scores); although, PRP showed sustained minimal clinically important benefit (MCID) at 6 months (p < 0.001). In the RCT published by Wongjarupong et al. [18], it was demonstrated that transforaminal epidural injection of PRP demonstrated clinical meaningful improvements (significant MCID) in leg VAS score at 6, 12, and 24 weeks; and in ODI at 24 weeks. They concluded that epidural, double-spin PRP provided significantly better results in comparison with triamcinolone administration.

On the other hand, in the RCT by Xu et al. [22], among 124 patients treated with USG-guided TF epidural injections using steroid or PRP, similar outcome was observed between the two patient groups in terms of VAS, ODI, and SF-36 (physical function and bodily pain) and complications at various time points (1 week, 1 month, 3 months, 6 months, and 1 year). Thus, based on the previously published RCTs, the reporting of the improvement in pain and disability is substantially varied across different time points [18, 21, 22, 24, 25]. While some studies have reported better early or more consistent outcome with PRP, others have observed no significant differences between the two approaches.

### Corroborative evidence from our meta-analysis

For our analysis, we compared the outcome and complications reported at 1-, 3-, 6-, 12- and 24-week time points in the reviewed articles. We compared the outcome based on the VAS, ODI and SF-36 scores, as reported in the reviewed RCTs. Based on our analysis, we did not observe any significant difference between the two groups (ESI vs. epidural PRPs) at all the time points for all the aforementioned outcome measurements. Thus, the current evidence does not demonstrate a substantial superiority of PRP injections over ESI, in terms of clinical effectiveness or efficacy (either during the early or delayed post-intervention time points). Among the studies, only Saraf et al. [24] included a clinical parameter (straight leg raising test - SLR) for evaluating the outcome during the followup assessments. In this study, at the end of 6 months, 90% and 62% of patients were SLR (straight leg raising) test negative in the PRP and steroid groups, respectively.

### Complication rates

Among the reviewed RCTs, only 2 major complications were directly attributed to the medications. In the study by Ruiz Lopez et al. [21], one male patient in the leucocyte-rich PRP group experienced itching in the pelvic area, which was relieved following treatment with antihistaminics. In the another RCT [18], 2 patients in the triamcinolone injection group required to undergo surgery within 6 weeks in view of treatment failure. Our metaanalysis also (similar to other previous reviews [22–24, 30, 49, 50]) did not reveal any statistical difference in the complication or adverse event rates between the two interventions.

In the RCT by Gupta et al. [25], they concluded that since PRPs are autologous components, multiple injections could be safely administered (with minimal added complications), as compared to corticosteroids. Thus, based on the available literature, there is significant evidence to support the safety of epidural PRP injections in patients with lumbar radiculopathy.

### Evidence based on other systematic reviews on PRP

Overall, pain intensity has been variously evaluated in the literature using different parameters including VAS score, numerical pain score (NPS), Lattinen index, COMI pain score (CPS) and Oswestry pain score (OPS); functional outcome by Oswestry Disability Index (ODI), functional rating index (FRI), single assessment numerical evaluation (SANE), MacNab criteria, Modified Oswestry Disability Questionnaire (MODQ), physical performance test (PPT), SF-36 with subscores for bodily pain and physical functioning, COMI disability score (CDS) and Rolland Morris Disability Questionnaire (MODQ) [21, 28, 29, 45].

Although a majority of the systematic reviews hitherto published [23, 28, 29], all the studies have highlighted on the lack of high quality and reliable evidence on this subject. In a recent systematic review, Machado et al. [29] concluded that a majority of published studies have revealed positive results regarding the effectiveness of PRP, with a relatively low risk of overall bias. The quality of evidence supporting PRP in LBP was graded as level-II (defined as “moderate evidence from at least one relevant high-quality RCT or multiple relevant moderate-/lower-quality RCTs). They emphasized on the need for large-scale, multi-centered RCT on this subject to further substantiate their observations.

In another systematic review by Kubrova et al. [28], 12 studies (3 RCTs and 9 observational studies) were considered. While pain intensity was the primary outcome evaluated; functional improvement, radiological findings and complications were the secondary outcome parameters considered. They also demonstrated that PRP was associated with similar or longer pain relief, with effects extending up to 12 or 24 months. The Grading of Recommendations Assessment, Development and Evaluation (GRADE) analysis showed very low certainty of available evidence owing to the risk of bias and imprecision. We therefore conducted a meta-analysis including only RCTs (2 additional RCTs have been published since the last review) in order to provide a more definite verdict on this subject.

### Limitations of our study

Only 5 RCTs have been published on this subject hitherto, with relatively small sample size. Although a wide variety of outcome measures have been examined in the literature heretofore; only certain parameters (VAS, ODI and SF-36) were amenable to meta-analysis. There are variations in the PRP preparations, techniques for injections, post-intervention protocols and follow-up strategies employed in the individual studies. Nevertheless, our study is the only meta-analysis to provide the highest quality evidence, based only upon the updated RCTs published till date on this issue.

## Conclusion

Epidural administration of PRP offers comparable (and not superior) benefit as ESI in the management of radiculopathy due to LDD. The safety profile of the epidural PRP is also similar to ESI. Nevertheless, large-scale, multi-centric RCTs involving larger sample population, and longer follow-up are necessary to further validate our observations.
