# Supplementary material for: Is platelet-rich plasma better than steroids as epidural drug of choice in lumbar disc disease with radiculopathy? Meta-analysis of randomized controlled trials
Source: Exp Biol Med (Maywood). 2025 Feb 4;250:10390. doi: 10.3389/ebm.2025.10390 (PMC11832311; doi:10.3389/ebm.2025.10390)
Supplement: Supplementary file 1 [file DataSheet1.docx]

Supplementary Table 1: General characteristics of the included studies.

| Study No | Author | Year | Country | Study Design | Sample size | | Age | | Male : Female | | Levels | | Outcomes | Follow-up |
| --- | --- | --- | --- | --- | --- | --- | --- | --- | --- | --- | --- | --- | --- | --- |
|  |  |  |  |  | PRP group | Steroid group | PRP group | Steroid group | PRP group | Steroid group | PRP group | Steroid group |  |  |
| 1 | A Gupta | 2024 | India | RCT | 23 | 23 | 40.64 | 38.92 | 14:09 | 17:06 | L4-L5 12; L5-S1 11 | L4-L5 12; L5-S1 11 | mODI, VAS, SF-12 | 1 year |
| 2 | A Saraf | 2023 | India | RCT | 29 | 31 | 42.03 | 45.83 | 15:14 | 16:15 | L3-L4 2; L4-L5 20; L5-S1 7 | L3-L4 0; L4-L5 24; L5-S1 6 | mODI, VAS, SLRT, Failures | 6 months |
| 3 | A Wongjarupong | 2019 | Thailand | RCT | 15 | 15 | 39.73 | 39.13 | 9:6 | 8:7 | L4-L5 8; L5-S1 7 | L4-L5 7; L5-S1 8 | mODI, VAS, Adverse Event, Failures | 6 months |
| 4 | R Ruiz-Lopez | 2020 | Spain | RCT | 25 | 25 | 68 | 61 | 11:14 | 10:15 | NA | NA | VAS, SF-36 | 6 months |
| 5 | Z Xu | 2021 | China | RCT | 61 | 63 | 56 | 56 | 28:33 | 37:26 | NA | NA | mODI, VAS, SF-36, F-wave rate & latency | 1 year |

NA – Not Available; mODI – modified Oswestry Disability Index; PRP – Platelet-rich Plasma; RCT – Randomized Controlled Trial

Supplementary Table 2: Intervention Protocols of included studies.

| Study No | Author | PRP intervention | Steroid intervention | Route of administration | Outcome measures |
| --- | --- | --- | --- | --- | --- |
| 1 | A Gupta | 2ml PRP with 0.5ml 0.5% Bupivacaine | 40mg Triamcinolone with 0.5ml 0.5% Bupivacaine | Fluoroscopy-guided transforaminal epidural route | mODI, VAS, SF-12 |
| 2 | A Saraf | 3 ml of autologous PRP | 2 ml of methylprednisolone acetate (40 mg/ml) with 1 ml 1% lignocaine | Fluoroscopy-guided transforaminal epidural route | mODI, VAS, SLRT, Failures |
| 3 | A Wongjarupong | 2 mL of PRP followed by NS 0.5 ml | 2ml of 1% lidocaine with 40 mg triamcinolone | Fluoroscopy-guided transforaminal epidural route | mODI, VAS, Adverse Event, Failures |
| 4 | R Ruiz-Lopez | 16.5 mL of LR-PRP | 20 ml with 60 mg of triamcinolone acetonide | USG-guided transforaminal epidural route | VAS, SF-36 |
| 5 | Z Xu | 3 ml autologous PRP | 2 ml betamethasone + 0.5 ml 0.9% saline + 0.5 ml 2% lidocaine | Fluoroscopy-guided caudal epidural route | mODI, VAS, SF-36, F-wave rate & latency |
